# Comparative metabolomics profiling of engineered *Saccharomyces cerevisiae* lead to a strategy that improving β-carotene production by acetate supplementation

**DOI:** 10.1371/journal.pone.0188385

**Published:** 2017-11-21

**Authors:** Xiao Bu, Liang Sun, Fei Shang, Guoliang Yan

**Affiliations:** 1 Center for Viticulture and Enology, College of Food Science and Nutritional Engineering, China Agricultural University, Beijing, P.R., China; 2 Key Laboratory of Viticulture and Enology, Ministry of Agriculture, Beijing, P.R., China; 3 Beijing Key Laboratory of Bioprocess, Beijing University of Chemical Technology, Beijing, P.R., China; University of Szeged, HUNGARY

## Abstract

A comparative metabolomic analysis was conducted on recombinant *Saccharomyces cerevisiae* strain producing β-carotene and the parent strain cultivated with glucose as carbon source using gas chromatography-mass spectrometry (GC-MS), high performance liquid chromatography-mass spectrometry (HPLC-MS) and ultra-high performance liquid chromatography-tandem mass spectrometry (UPLC-MS/MS) based approach. The results showed that most of the central intermediates associated with amino acids, carbohydrates, glycolysis and TCA cycle intermediates (acetic acid, glycerol, citric acid, pyruvic acid and succinic acid), fatty acids, ergosterol and energy metabolites were produced in a lower amount in recombinant strain, as compared to the parent strain. To increase β-carotene production in recombinant strain, a strategy that exogenous addition of acetate (10 g/l) in exponential phase was developed, which could enhance most intracellular metabolites levels and result in 39.3% and 14.2% improvement of β-carotene concentration and production, respectively, which was accompanied by the enhancement of acetyl-CoA, fatty acids, ergosterol and ATP contents in cells. These results indicated that the amounts of intracellular metabolites in engineered strain are largely consumed by carotenoid formation. Therefore, maintaining intracellular metabolites pool at normal levels is essential for carotenoid biosynthesis. To relieve this limitation, rational supplementation of acetate could be a potential way because it can partially restore the levels of intracellular metabolites and improve the production of carotenoid compounds in recombinant *S*. *cerevisiae*.

## Introduction

**Fig 1:**
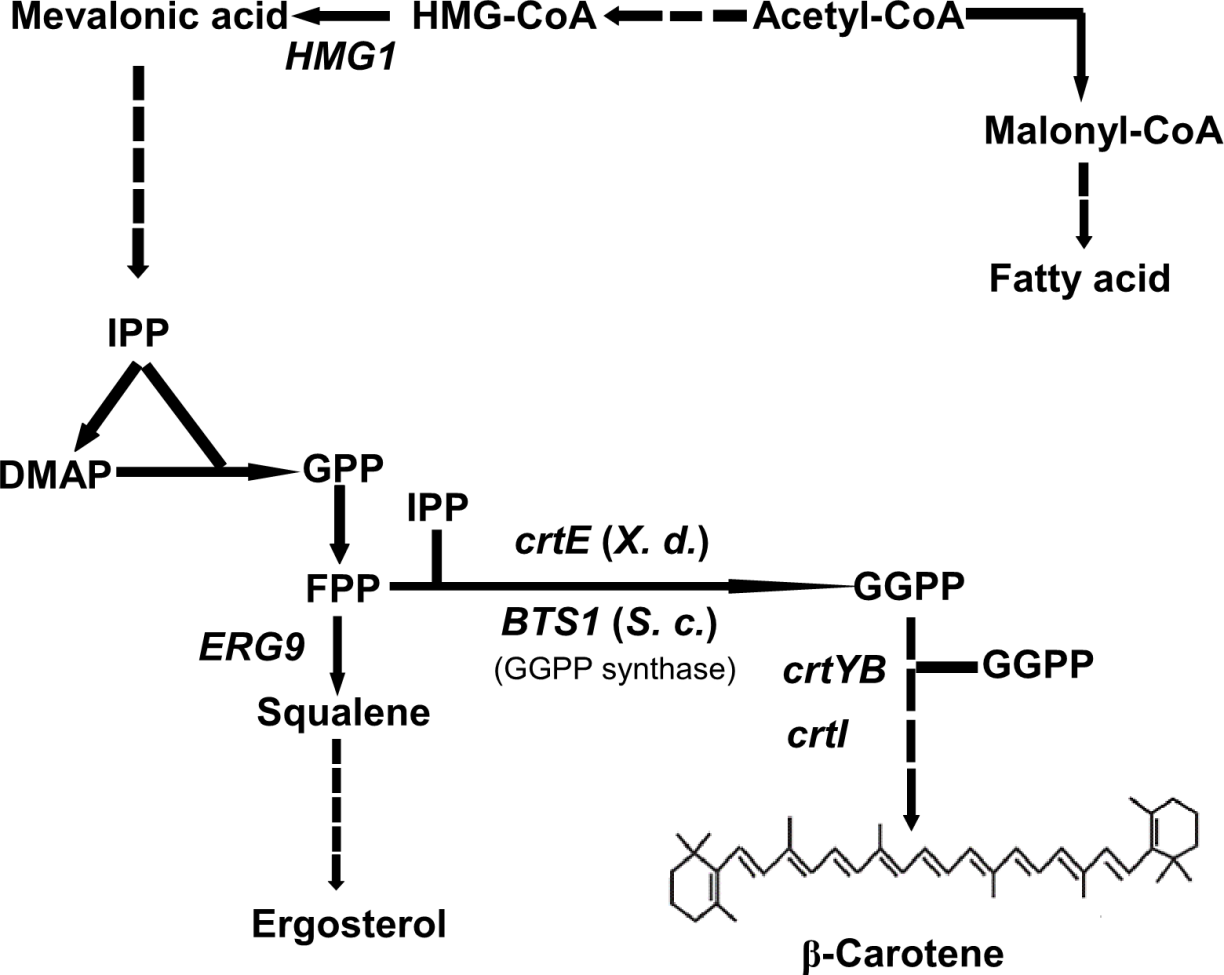
Biosynthetic pathway of β-carotene in recombinant *S*. *cerevisiae*. The dashed lines indicate multiple step reactions. *HMG1*: HMG-CoA reductase, *ERG9*: squalene synthase. Carotenoids pathway: *CrtE*: GGPP synthase, *BTS1*: *S*. *cerevisiae* GGPP synthase, *CrtYB*: Lycopene cyclase, *CrtI*: Phytoene desaturase. IPP: isopentenyl pyrophosphate; DMAP: dimethylallyl pyrophosphate; GPP: geranyl pyrophosphate; FPP: farnesyl pyrophosphate; GGPP: geranylgeranyl pyrophosphate.

Carotenoids are natural pigments synthesized by plants and microorganisms [1]. Beta-carotene is an orange-colored carotenoid that possesses powerful free radical quenching activity, which has lots of applications in pharmaceuticals, neutraceuticals, cosmetics and foods [2]. Nearly 90% commercialized β-carotene is currently produced through chemical synthesis, however, its production by microbial fermentation had increased interests due to the restricted rules and regulations applied to obtaining chemicals [3,4]. Recently, carotenoids have been successfully synthesized in non-carotenogenic microorganisms such as *Escherichia coli* [5] and *Saccharomyces cerevisiae* [6]. In *S*. *cerevisiae*, heterologous β-carotene is synthesized via the mevalonate (MVA) pathway. The precursor acetyl-CoA is converted to isopentenyl pyrophosphate (IPP) and its isomer dimethylallyl pyrophosphate (DMAPP), which are transformed to geranylgeranyl pyrophosphate (GGPP) via the enzyme GGPP synthase. The introductions of three exogenous genes, *crtE*, *crtYB* and *crtI* drives two GGPP molecules into the carotenoid synthesis pathway to synthesizes β-carotene (Fig 1) [6,7]. To increase β-carotene production in engineered strain, a variety of metabolic engineering strategies have been developed, such as overexpression of the rate-limiting enzyme *HMG1* to enhance the flux of mevalonate pathway [6,8], increase cofactor (ATP and NADPH) supplies to provide extra energy for β-carotene production [5] or down-regulation of *ERG9* to limit ergosterol accumulation and drives additional farnesyl pyrophosphate (FPP) into carotenoid synthesis pathway [9]. Besides application of metabolic strategies, optimization of fermentation condition such as changing carbon source [10], controlling pH [11] and oxygen level [12] could also efficiently promote carotenoid production.

It is well known that when heterologous bio-compound is biosynthesis in an organism, the cell will change its metabolism and re-program the genomic expression as an adaptive response [13]. This phenomenon is pronounced in the heterologous production of carotenoids in *S*. *cerevisiae* because carotenoid formation can decrease functional compounds, like sterols, dolichols and unsaturated fatty acid due to sharing the common precursors (acetyl-CoA and FPP), which in turn can negatively influence cell growth and carotenoid production [14]. It has been demonstrated that revealing the transcriptional changes in strains producing carotenoids can help to identify the potential factors limiting the formation of carotenoids and design effective strategies to improve carotenoid production [15,16]. For example, Verwaal et al. [16] used DNA microarray to found that the heterologous biosynthesis of carotenoid in *S*. *cerevisiae* strain (CEN.PK113-7D) can induce multidrug-resistant transporter synthesis, encoded by pleiotropic drug-resistance genes (Pdr10), and concluded that this can facilitate the secretion of carotenoids to the environment to decrease the toxicity within the cells. Based on these results, Lee et al. [17] transformed Pdr10 from *S*. *cerevisiae* into *Rhodosporidium toruloides*, and found that Pdr10 strain cultivated in the two-phase media could obtain higher production of carotenoids due to continuous export of carotenoids in situ.

Metabolomic analysis is a powerful tool to observe comprehensive intracellular metabolite concentrations, which consists of the entire pool of low molecular weight metabolites in an organism including amino acids, amines, nucleotides, sugars, lipids, and other substances [18,19]. Due to the fact that metabolome data are the final downstream products of gene expression and cellular regulatory processes, they can better reflect the metabolic state of the cell than transcriptomic or proteomic data [18,19]. Metabolomic analysis has been successfully applied to identify the key metabolites required for stress tolerance [19,20]. As far as we know, comparative metabolomics analysis has not yet been performed for heterologous carotenoids production in yeast, which may provide vital information to design an effective strategy to improve carotenoid production. In this study, GC-MS, HPLC-MS and UPLC-MS/MS were used to determine the metabolic response of *S*. *cerevisiae* to heterologous β-carotene formation by comparing intracellular metabolite profiles of the recombinant strain and the parent strain. The results revealed that 47 central intermediates associated with amino acids, carbohydrates, glycolysis and tricarboxylic acid cycle (TCA) intermediates, fatty acids, ergosterol and energy metabolism were dramatically reduced by heterologous β-carotene biosynthesis. Consequently, a strategy that exogenous supplementation of acetate in exponential phase was developed, which can largely replenish most intracellular metabolites and increase β-carotene production. Our results suggested that metabolomics approach is a powerful tool to design the effective strategy to increase carotenoid production in microorganism.

## Materials and methods

### Yeast strains and plasmid

The industrial wine yeast T73-4 (MATa; ura3-52) [21] and the recombinant *S*. *cerevisiae* T73-63 [22] were used in this study. The strain T73-63 was derived from strain T73-4 by being transformed with the integration vectors YIplac211YB/I/E* (11,579 bp). This plasmid carrying the carotenoid biosynthesis genes was kindly presented by Verwaal [6], which includes the gene *crtYB* (encodes a bifunctional phytoene synthase and lycopene cyclase), *crtI* (phytoene desaturase) and *crtE* (heterologous GGPP synthase) cloned from *Xanthophyllomyces dendrorhous*. The expressions of three genes were driven by the *S*. *cerevisiae* GPD strong constitutive promoter and CYC1 terminator.

### Fermentation conditions

Yeast strains were pre-cultured aerobically in YPD medium (2% glucose, 2% peptone and 1% yeast extract) at 30°C and 200 rpm for approximately 15 h up to the late exponential phase. Then the seed culture was inoculated into 500 ml shake flask containing 200 ml YPD medium to an initial optical density (OD_600_) of 0.1 and continuously agitated (180 rpm) at 30°C. According to our previous study [11], pH 6.0 is optimal for β-carotene production. Therefore, the initial pH of the YPD medium was adjusted to approximately 6.0. In the fermentation process, the pH value varied between 5.5 and 6.0; otherwise, it was adjusted with 3 M NaOH or 3 M H_2_SO_4_. Samples were collected at the indicated time points and analyzed for cell growth, β-carotene and metabolites concentrations. All experiments were independently repeated three times, and data in the figures and table are expressed as the averages ± standard deviations. Statistical significance (*P*<0.05) was determined by Student’s *t*-tests.

### Analysis and quantification of main metabolites and β-carotene

Yeast growth was determined via the absorbance of OD_660_ and dry weight of biomass as described in our previously article [23]. The concentrations of glucose, ethanol and acetic acid were measured via HPLC using a Bio-Rad HPX-87H column, as described in the previous study [24]. Carotenoids were extracted from the cultured cells with acetone-methylene chloride (50:50, v/v) and used for analysis via HPLC, as described previously [7]. The concentration of β-carotene was calculated in milligrams per extracted gram dry cell weight (DCW).

### Metabolite extraction and derivatization

Samples for metabolome analysis were prepared as described in detail previously [18]. The dried yeast cell debris was suspended in pyridine (50 μl) and sonicated at 37°C for 60 min to break the cell wall and release metabolites from cells. Subsequently, 50 μl methoxyamine-HCl (20 mg/ml in pyridine) was added then placed in a water bath at 80°C for 30 min. Silylation was then carried out by adding 99 μl of N-methyl-N-(trimethylsilyl) trifluoroacetamide (MSTFA) with 1 μl of trimethylchlorosilane (TMCS) and incubated for 30 min at 70°C. After centrifugation (4,000 rpm, 5 min, room temperature), samples were transferred to an insert in an autosampler vial and analyzed by GC-MS.

### Metabolite analysis with GC-MS

An Agilent GC-MS 7890B-5977A system (Agilent Technologies, Santa Clara, CA, USA) was used for sample analysis, as described in detail previously and modified [18]. The derivatized samples (1 μl) were injected via a HP-5ms column (30 m×0.25 mm, 0.25 μm film thickness) using pulsed splitless injection (25 psi for 30 s). The split vent was opened after 60 s. After 60°C hold for 1 min, the column oven temperature was increased at 10°C min^-1^ to 325°C, and held at 325°C for 10 min. High purity helium was used as carrier gas with a constant flow rate of 1 ml min^-1^. The mass spectrometer was operated in electron impact (EI) mode at 70 eV and in the scan and selective ion mode (SIM) range of 20–350 m/z. The mass spectra and retention time obtained were used to identify the metabolites.

### Amino acids and energy metabolism analysis

For the precise analysis of the intracellular content of amino acids, Agilent 1100 series HPLC-MS system with BEH Amide (1.7 μM 100×2.1 mm, Waters) column was conducted. Remove the sample from -80°C, add 500 μl methanol immediately, then 2000 rpm shock for 1 h. The supernatant was removed by centrifugation, then add 500 μl of water, 2000 rpm shock for 1 h. Centrifuge the supernatant and merge with the previous supernatant. Dried in refrigerated vapor traps (Thermo Scientific Savant RVT5105), and dissolved with 100 μl of 50% methanol, then transferred to an insert in an autosampler vial and analyzed by HPLC-MS. Detection and identification of amino acids is achieved using 5500 QTRAP tandem mass spectrometer, operated in selective MRM mode. The flow rate was 0.3 ml/min, injection volume was 1 μl and column temperature was 50°C. The solvents were (A) acetonitrile containing 2.5 mmol/l ammonium formate and formic acid (0.1%) and (B) water containing formic acid (0.1%) with a gradient over 38 min as follows: 95%A+5%B for 2 min, 50%A+50%B for 14 min, and 95%A+5%B for 22.1 min. For quantification, a standard calibration curve was constructed with at least seven solutions of defined amounts of amino acids (Sigma-Aldrich).

The analysis of intracellular energy metabolites was performed by UPLC-MS/MS (Thermo Scientific^TM^ Ultimate 3000^TM^ Q Extractive^TM^) equipped with ACQUITY UPLC HSS T3 C18 column (1.7 μm, 2.1×100 mm, Waters), the pretreatment of samples was same with HPLC-MS. Water containing 0.1% formic acid (A) and acetonitrile (B) were used as the mobile phase with a flow rate of 0.4 mL/min at 40°C. Elution program as follows: 100%A for 2 min, 100%B for 8min and 100%A for 3 min. For quantification, a standard calibration curve was constructed with at least seven solutions of defined amounts of energy metabolites (Sigma-Aldrich).

## Results

### Cell growth, glucose consumption and β-carotene production of recombinant strain T73-63

**Fig 2:**
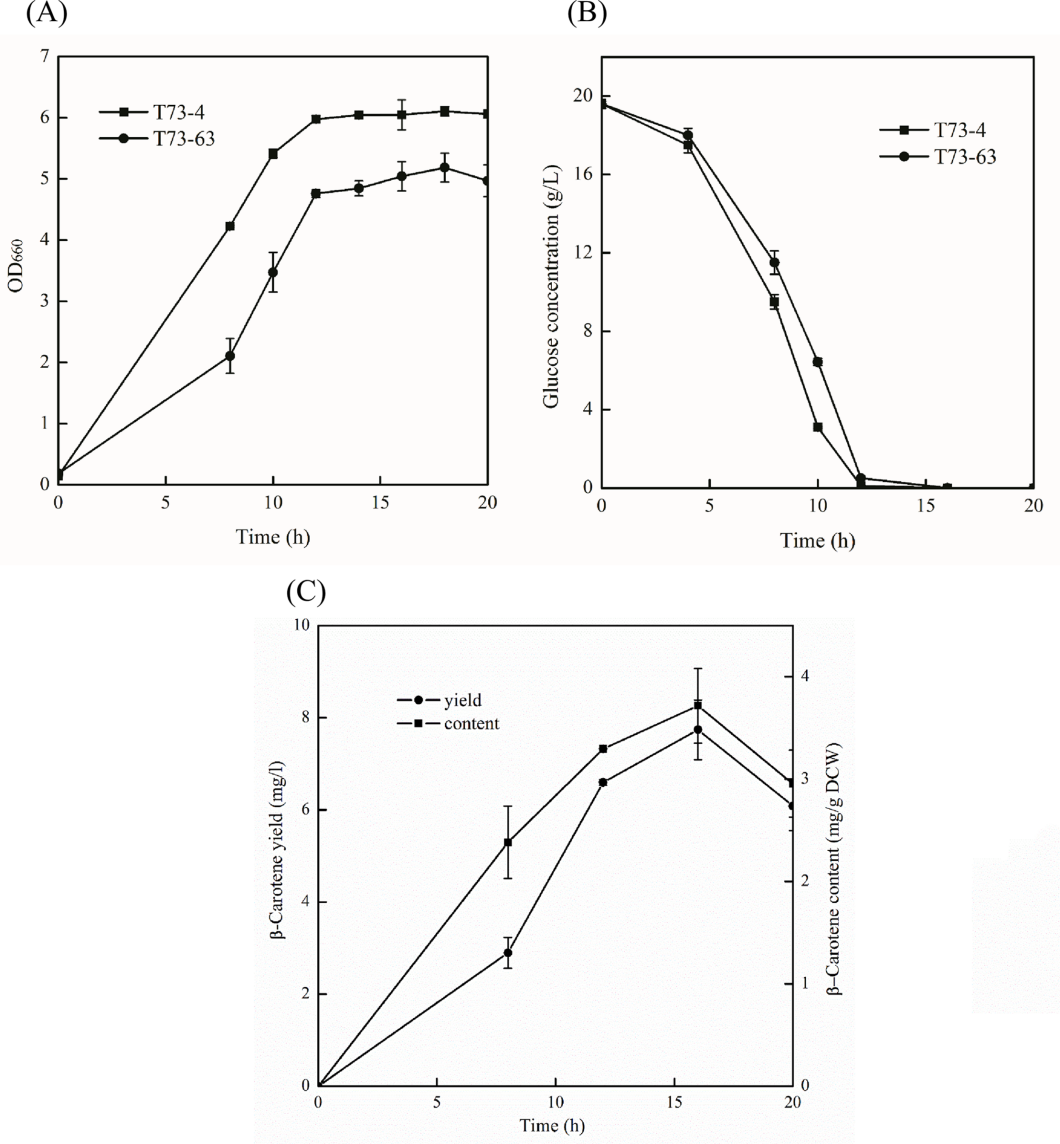
**Cell growth (A) and glucose consumption (B) of recombinant *S*. *cerevisiae* T73-63 and parent strain T73-4 and β-carotene concentration and production (C) of recombinant *S*. *cerevisiae* T73-63.** Values represent the average of three independent cultures, and error bars correspond to the standard deviation (*P*< 0.05, Student’s *t* test).

As previously mentioned, the biosynthesis of heterologous bio-compounds can exert a remarkable effect on cells growth and metabolism. Thus, the cell growth, glucose consumption and β-carotene production of recombinant strain T73-63 and the parent strain T73-4 were determined (Fig 2). The growth periods of two strains were consistent and could be divided into 3 phases: log phase (0–6 h), exponential phase (6–12 h) and stationary growth phase (after 12 h). As expected, the cell growth (Fig 2A) and glucose consumption (Fig 2B) of recombinant strain were negatively influenced by β-carotene biosynthesis compared to the parent strain due to metabolic burden, which was consistent with previous literature data [22,25]. The β-carotene concentration and production gradually increased with fermentation time, and the highest values (3.72 mg/g dry cell weight and 7.74 mg/l broth) were obtained at 16 h in recombinant strain T73-63 (Fig 2C).

### Metabolite profiles of the recombinant strain T73-63 and the parent strain T73-4

**Fig 3:**
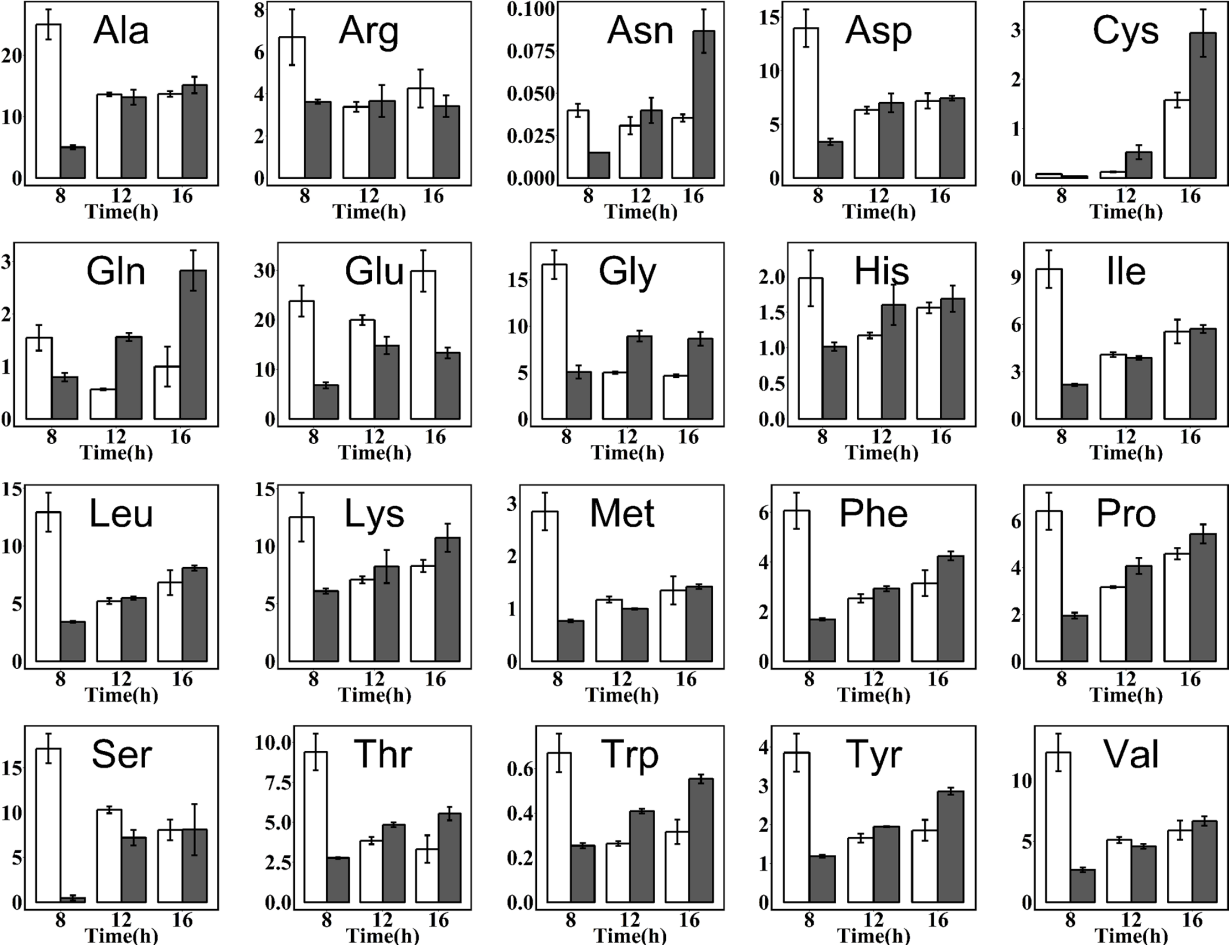
Time courses of intracellular amino acids concentration (μmol/g) of recombinant *S*. *cerevisiae* T73-63 (marked grey) and parent strain T73-4 (marked white). Values represent the average of three independent cultures, and error bars correspond to the standard deviation (*P*< 0.05, Student’s *t* test).

**Fig 4:**
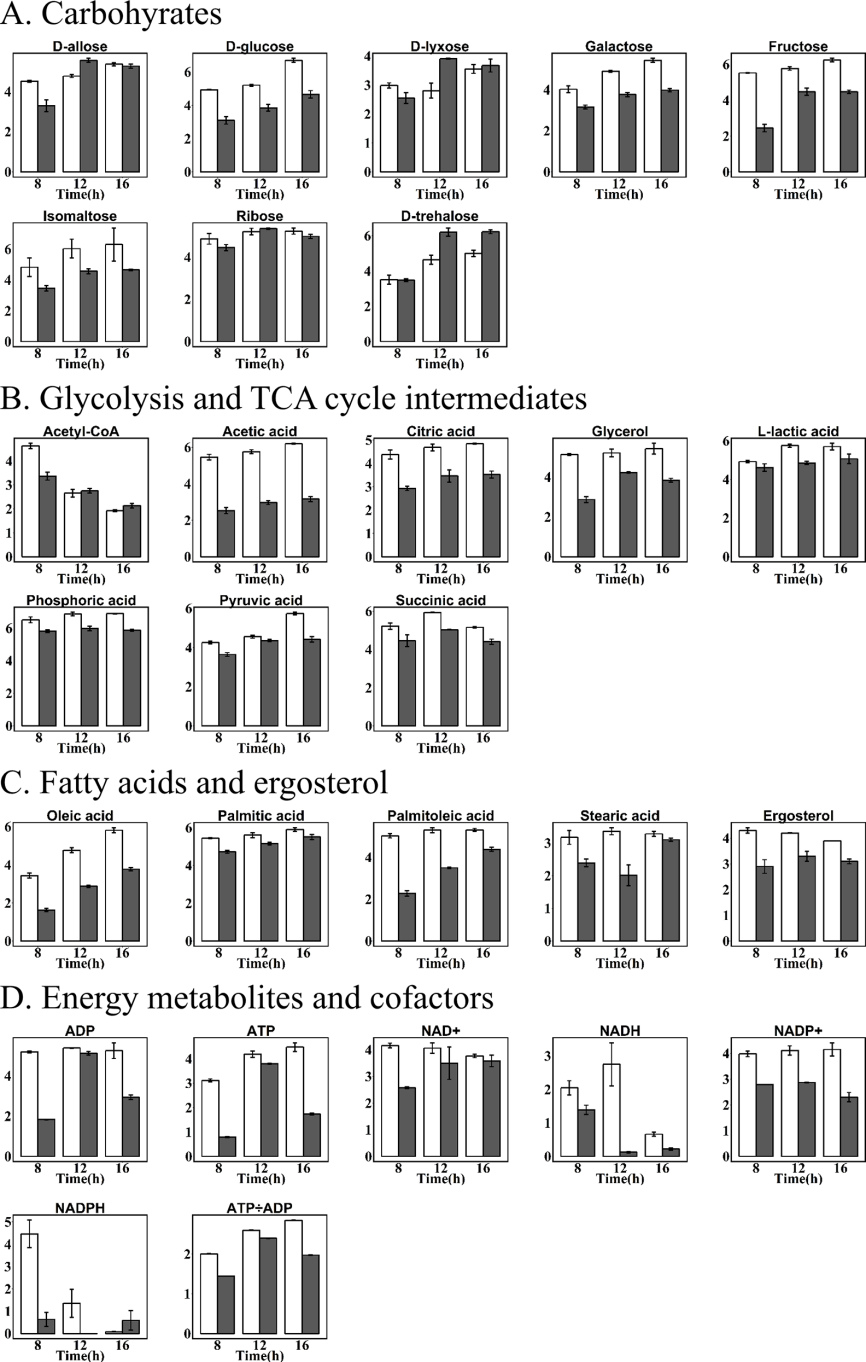
Time courses of relative concentration of metabolites in recombinant *S*. *cerevisiae* T73-63 (marked grey) and parent strain T73-4 (marked white). Values represent the average of three independent cultures, and error bars correspond to the standard deviation (*P*< 0.05, Student’s *t* test).

A time-dependent metabolomics approach was applied to understand its metabolism for carotenoid production between two strains at three different time points (8, 12 and 16 h) representative of early exponential, late exponential and stationary growth phase. A total of 82 metabolites were identified by GC-MS, HPLC-MS and UPLC-MS/MS (S1 Table), the metabolite profiles revealed 47 central intermediates associated with amino acids, carbohydrates, glycolysis and TCA cycle intermediates, fatty acids, ergosterol, cofactors (NAD^+^, NADH, NADP^+^ and NADPH) and energy metabolites (ATP and ADP) (Figs 3 and 4). Generally, heterologous carotenoids accumulation in yeast reduced the contents of these intermediates, while it was associated with cultivation time.

In *S*. *cerevisiae*, amino acids such as threonine, serine, glycine, alanine, glutamate and proline can be synthesized from compounds in the glycolysis pathway and TCA cycle. The contents of amino acids in recombinant strain T73-63 were significantly varied compared to the strain T73-4 dependent on the culture time (Fig 3). At early exponential phase (8 h), all determined amino acid levels were significantly lower in recombinant strain than those of the parent strain, but this reduction gradually disappear when cells enter the exponential phase. Conversely, some of amino acids contents became higher than those of the parent strain, especially asparagine, cysteine and glutamine. This might suggested that less amino acids were synthesized by cells due to carotenoid biosynthesis during early fermentation phase, which redirect more available acetyl-CoA to enter the carotenoid synthesis pathway, while this demand decreased in the stationary growth phase, as shown in Fig 4B.

Carbon source is a crucial factor for carotenoid biosynthesis [26]. In this work, we found that the relative contents of glucose, fructose, galactose and isomaltose in recombinant strain were all lower than those in parent strain at three analyzed time points (Fig 4A). Especially glucose and fructose, their contents was 37.3%, 30.4% and 55.7%, 28.4% lower than those of the parent strain at 8 h and 16 h, respectively. Interestingly, higher amount of trehalose was observed to be accumulated in recombinant strain T73-63 at 12 h and 16 h since their contents were 33.8% and 24.4% higher than those of the parent strain T73-4, respectively. Similar results were reported by Martinez-Moya et al. [10] that a simultaneous increment of trehalose was observed in succinate cultures with high astaxanthin production of *X*. *dendrorhous*. The TCA cycle is a key metabolic pathway in all cells. The main TCA cycle intermediate metabolites including lactic acid, acetic acid, citric acid, pyruvic acid and succinic acid were significantly changed in recombinant strain (Fig 4B). Similar to carbon source, a general down-regulation in production of the TCA cycle intermediates were found as compared to the parent strains. Especially acetic acid, its content was 46.4%, 51.8% and 52.3% lower than those of the strain T73-4 at 8, 12 and 16 h, respectively. We also found that acetyl-CoA level, the first precursor in mevalonate pathway for carotenoid biosynthesis, was significantly reduced after 8 h fermentation, although the contents of 12 and 16 h were slightly higher than those of the parent strains.

The main fatty acids synthesized by *S*. *cerevisiae* were palmitic acid (C16:0), stearic acid (C18:0), palmitoleic acid (C16:1) and oleic acid (C18:1) [27]. There is competition for acetyl-CoA between carotenoid and fatty acid branch. Our previous studies indicated that heterogeneous biosynthesis of carotenoid in *S*. *cerevisiae* led to a decreased contents of fatty acids in cells, especially unsaturated fatty acids, palmitoleic acid and oleic acid [23]. Similar results were observed in this study (Fig 4C), the concentrations of oleic acid at 8 h, 12 h and 16 h were 52.8%, 39.7% and 35.2% lower than those of control, respectively. In the case of palmitoleic acid, the values were 54.6%, 34.0% and 17.5%, respectively. There is also competition for FPP between carotenoid and ergosterol branch. The changes of ergosterol content in both strains were therefore determined, and found that its concentration in recombinant strain were 32.6%, 21.4% and 20.5% lower than those of the control at 8 h, 12 h and 16 h, respectively. These results confirmed that heterologous carotenoid biosynthesis in yeast can drive more carbon source (such as acetyl-CoA and FPP) from the branch pathways of fatty acid and ergosterol to carotenoid pathway [15].

ATP and NADPH involved in energy metabolism are two important cofactors for β-carotene production [5]. We also found that the concentrations of cofactors including NAD^+^, NADH, NADP^+^ and NADPH in T73-63 strain were significantly lower than those in T73-4 strain. In addition, ADP, ATP level and the ATP/ADP ratio in T73-63 at 8 and 16 h were also significantly lower compared to those of T73-4 (Fig 4D). These results suggested that energy status in the recombinant strain became limited compared to the parent strain due to heterologous β-carotene biosynthesis in yeast.

### Effects of different carbon sources supplementation on cell growth and β-carotene biosynthesis in recombinant strain T73-63

As shown in Figs 3 and 4, most intracellular metabolites in recombinant strain were decreased compared to the parent strain. Since the biosynthesis of amino acid and TCA cycle intermediates is closely associated with acetyl-CoA formation and acetyl-CoA is an initial precursor to the formation of carotenoid in yeast, we believed that carotenoid biosynthesis consume more intermediates (such as acetic acid, citric acid, pyruvic acid, succinic acid and glycerol) to form acetyl-CoA, which resulted in the reduction of amino acid and TCA cycle intermediates produced in cells. Accordingly, exogenous supplementation of UFAs could efficiently promote carotenoid production due to saving more available acetyl-CoA for carotenoid biosynthesis [23]. Thus, we assumed that exogenous supplementation of specific carbon source and TCA cycle intermediates could make up the deficiency in cells and may improve cell growth and (or) carotenoids synthesis. To prove this hypothesis, the effects of adding different carbon sources on cell growth and carotenoids synthesis of recombinant strain T73-63 were investigated, including supplementation of fermentable carbon sources (glucose and galactose) and non-fermentable carbon sources (acetic acid, glycerol and citric acid). Direct supplying acetic acid and citric acid can lead to intracellular pH drop and generate acid stress; sodium acetate and sodium citrate were therefore used in this experiment. The recombinant strain T73-63 was cultured in YPD medium containing 1% glucose as carbon source supplemented with 4% glucose, 1% glycerol, 1% galactose, 1% sodium acetate and 1% sodium citrate (w/v), respectively, and pH in all medium was controlled around 6.0 during the fermentation. The YPD medium containing 2% glucose (w/v) was set as the control.

**Fig 5:**
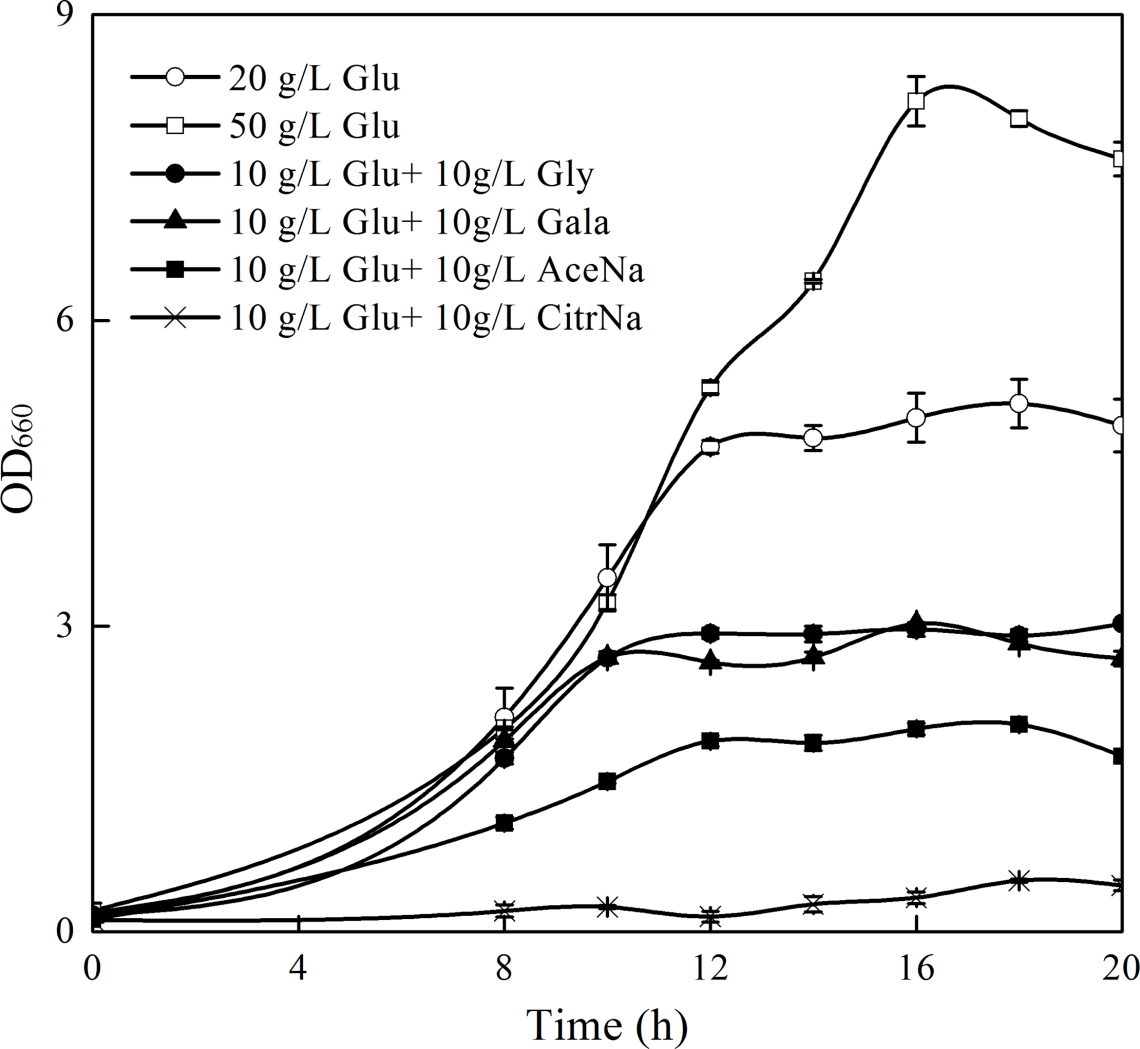
Cell growth of recombinant *S*. *cerevisiae* T73-63 in mixed carbon source cultures and YPD medium containing 2% and 5% glucose, respectively. Values represent the average of three independent cultures, and error bars correspond to the standard deviation (*P*< 0.05, Student’s *t* test). Abbreviations: Glu: glucose, Gly: glycerol, Gala: galactose, AceNa: sodium acetate, CitrNa: sodium citrate.

**Fig 6:**
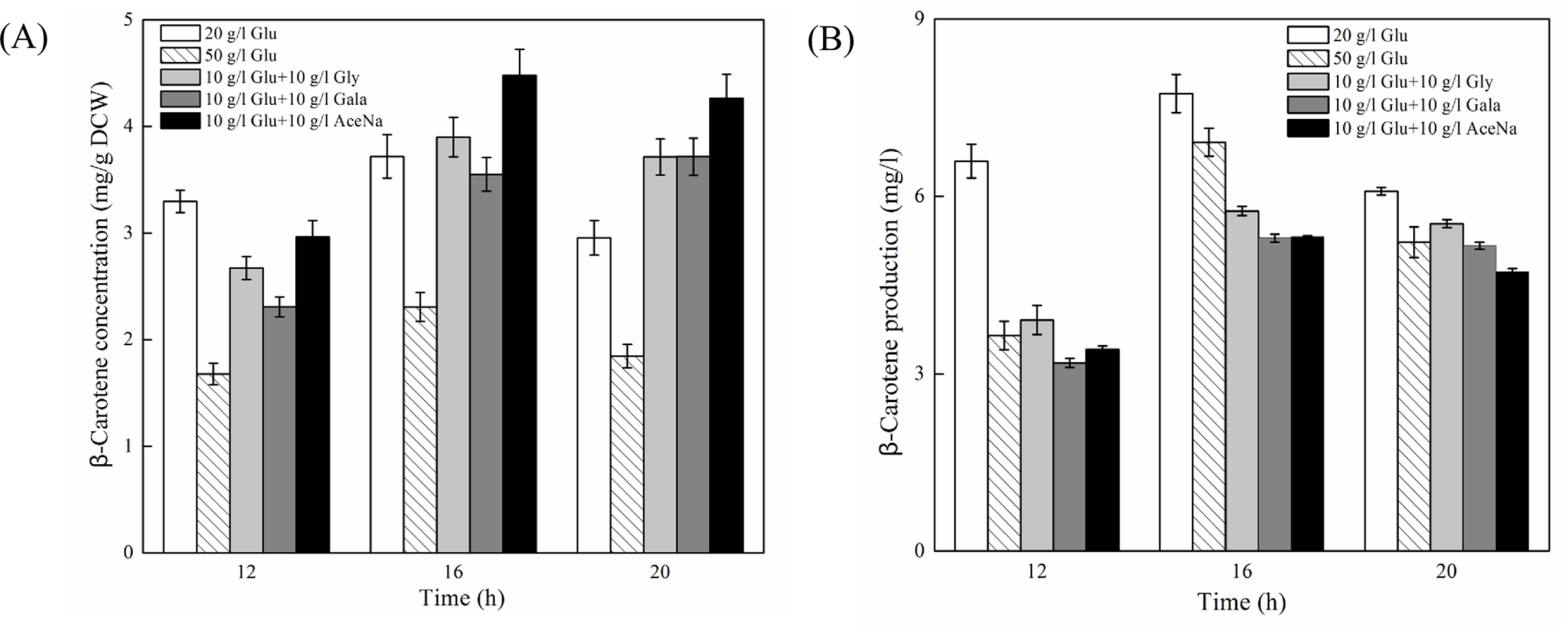
**β-Carotene concentration (A) and production (B) of recombinant *S*. *cerevisiae* T73-63 in mixed carbon source cultures and YPD medium containing 2% and 5% glucose, respectively at 12, 16 and 20 h.** Values represent the average of three independent cultures, and error bars correspond to the standard deviation (*P*< 0.05, Student’s *t* test). Abbreviations: Glu: glucose, Gly: glycerol, Gala: galactose, AceNa: sodium acetate.

Fig 5 showed that the highest biomass was obtained in medium containing 50 g/l glucose, which was 61.6% higher than that of the control containing 20 g/l glucose (16 h). In contrast, mixed carbon sources significantly inhibit cell growth. The maximum biomass in medium with glycerol, galactose and sodium acetate supplementation were 41.1%, 40.0% and 60.5% lower than that of control at 16 h, respectively. No apparent growth was observed in sodium citrate addition, suggesting that citrate is not a good carbon source for yeast growth, or may need to further optimize its added concentration or time. As regarding to β-carotene production, sodium acetate addition could achieve the highest β-carotene concentration (20.5% increment) after 16 h fermentation, followed by glycerol (4.9% increment) compared to that of control, respectively (Fig 6A). However, due to the strong inhibition on cell growth, the production of β-carotene with sodium acetate addition was only 68.7% of the control at 16 h (Fig 6B), implying that relieve the inhibition of cell growth is essential for enhancing carotenoid production. It should be noticed that high concentration of glucose (50 g/l) was not advantage for β-carotene biosynthesis, which could be ascribed to the fact that the transcription of genes in mevalonate pathway are subject to glucose repression [28].

### Effects of delaying acetate supplementation on cell growth, β-carotene production and intracellular metabolites profiles of recombinant strain T73-63

Initial addition of acetate could not effectively promote β-carotene production due to arrest of cell growth. Thus, we hypothesized that delay acetate added time, could yield higher β-carotene production due to relieving the inhibition of cell growth. The effects of adding 1, 2 and 10 g/l acetate after 10 h of cultivation (the time of cells entering late exponential phase) on β-carotene production were further investigated. Since relatively high glucose is beneficial for cell growth, we set the initial glucose concentrations in medium at 20 g/l. The cultivation in medium of 20 g/l glucose with initial addition of 10 g/l acetate was also performed.

**Fig 7:**
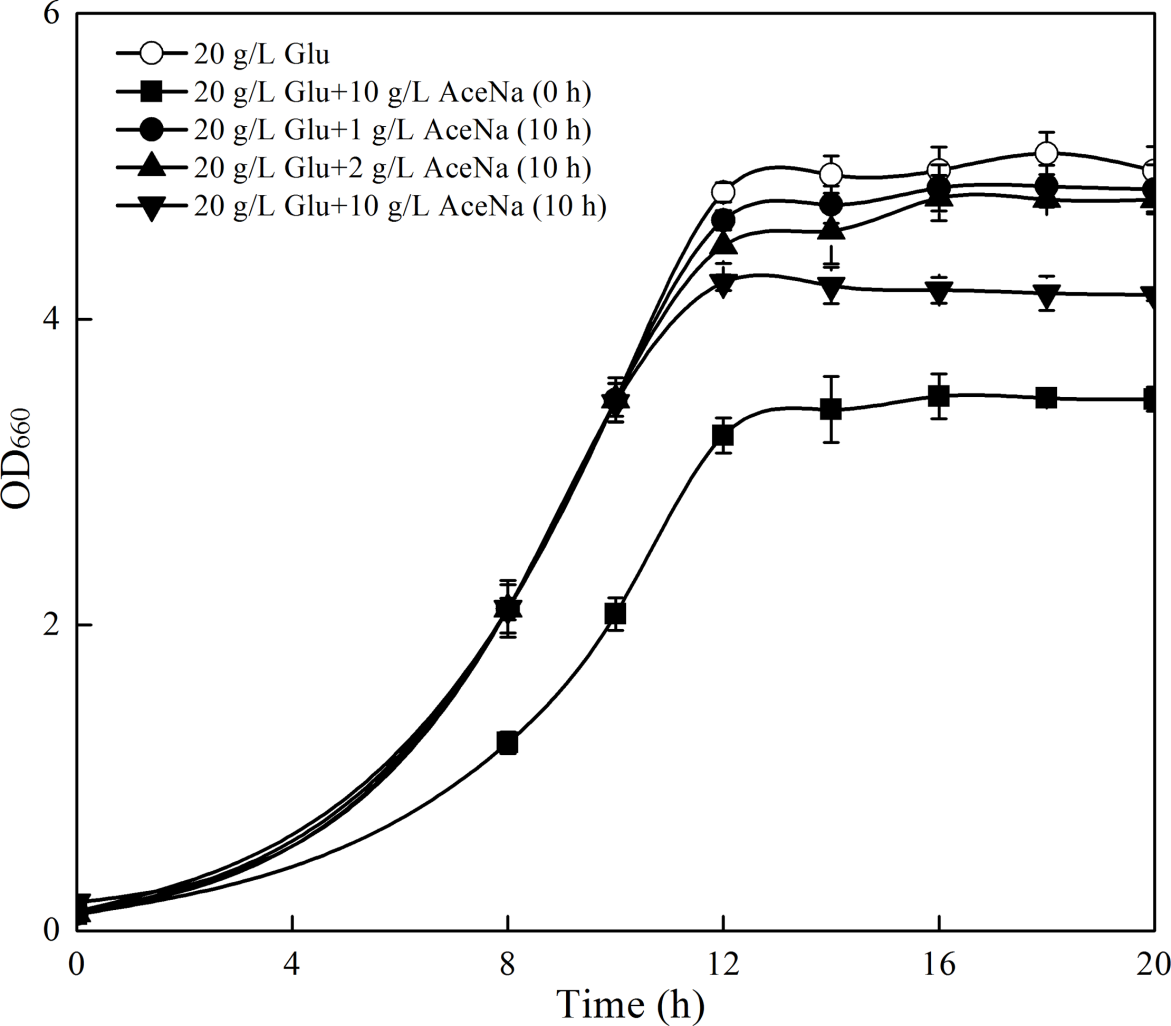
Cell growth of recombinant *S*. *cerevisiae* T73-63 with different acetate supplementation modes. Values represent the average of three independent cultures, and error bars correspond to the standard deviation (*P*< 0.05, Student’s *t* test). Abbreviations: Glu: glucose, AceNa: sodium acetate.

**Fig 8:**
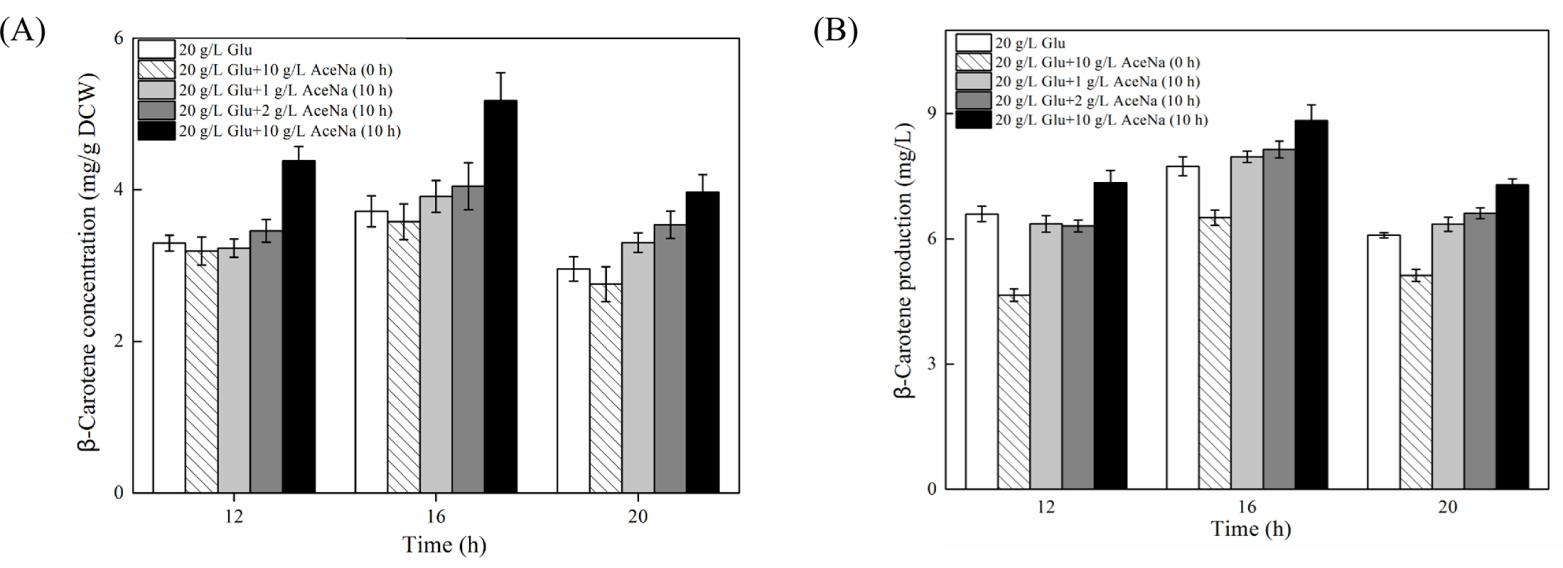
**The β-carotene concentration (A) and production (B) of recombinant *S*. *cerevisiae* T73-63 with different acetate supplementation modes.** Values represent the average of three independent cultures, and error bars correspond to the standard deviation (*P*< 0.05, Student’s *t* test). Abbreviations: Glu: glucose, AceNa: sodium acetate.

As expected, delaying sodium acetate addition reduced the inhibition on cell growth to some extent compared to initial addition, and the inhibition of cells became stronger with more sodium acetate added (Fig 7). However, the highest increment of β-carotene concentration was obtained in 10 g/l sodium acetate addition. The concentration of β-carotene with delaying 10 g/l acetate addition was increased to 4.38, 5.18 and 3.97 mg/g, which was 33.0%, 39.3% and 34.3% increment compared with the control without non-acetate supplementation at 12, 16 and 20 h, respectively (Fig 8A). As a result, the production of β-carotene was 11.4%, 14.2% and 20.0% higher than those of the control, respectively. The values were also 57.9%, 35.8% and 42.2% higher than those of initial acetate addition, respectively (Fig 8B).

To observe the effect of acetate on fermentation process, two kinetic parameters, including specific growth rate (*μ*cell) and specific sugar consumption rate (*q*s) in recombinant *S*. *cerevisiae* T73-63 and T73-63 supplemented with 10 g/l acetate at 0 h and 10 h were calculated by an interpolation method based on the data of S1A and S1C Fig. S1B and S1D Fig shows the change of *μ*cell and *q*s within the fermentation process. The concentration of ethanol and acetic acid were shown in supplementary data S1E and S1F Fig. The addition of acetate resulted in a decrease of cell growth rate and specific growth rate (S1A and S1B Fig), as well as glucose consumption and specific sugar consumption rate (S1C and S1D Fig). The production of ethanol was also inhibited by acetate (S1E Fig). With respect to acetic acid production, there is no remarkable change obtained in initial 10 g/l acetate addition. The control group without acetate addition increased the concentration of acetic acid by 0.45 g/l during 12 h and 20 h, and the acetic acid concentration with delaying 10 g/l acetate addition increased by 0.28 g/l, the specific acetic acid production rate of the latter was 29% lower than the former at 16 h, indicating that the addition of the acetate-treated groups were more likely to consume acetic acid than the control group and convert it into carotenoid synthesis pathway (S1F Fig).

**Fig 9:**
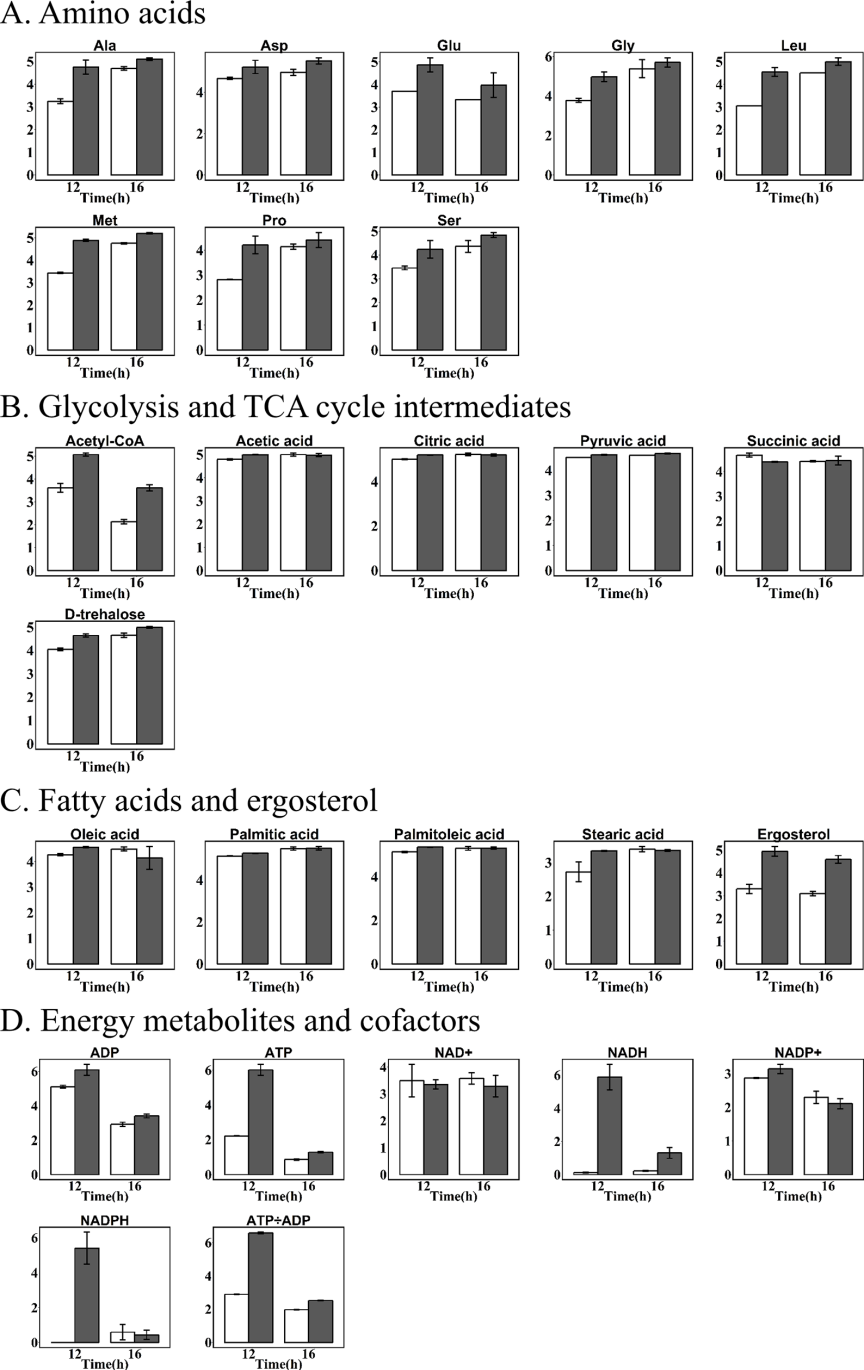
Relative concentration of metabolites in recombinant *S*. *cerevisiae* T73-63 supplemented with 10 g/l acetate at 10 h (marked grey) and T73-63 (marked white) at 12 and 16 h. Values represent the average of three independent cultures, and error bars correspond to the standard deviation (*P*< 0.05, Student’s *t* test).

We further compared the intracellular metabolites profiles with and without acetate supplementation after 10 h cultivation (S2 Table). The results showed that most of intracellular amino acids (especially alanine, glutamate, leucine and proline), TCA intermediates, fatty acids, ergosterol, cofactors and energy metabolites were replenished by acetate supplementation (Fig 9). Alanine, glutamate, methionine and serine, which were lower in recombinant strain than those of the parent strain T73-4 (Fig 3), were up-regulated as compared to those of the control at 12 h and 16 h (Fig 9A). The increased abundance of trehalose was also observed after acetate supplementation (Fig 9B). Except succinic acid, the TCA cycle intermediates (acetyl-CoA, acetic acid, citric acid, pyruvate), ergosterol and fatty acids (palmitic acid, stearic acid, palmitoleic acid and oleic acid) were also raised compared to the control, especially after 2 hours of addition (Fig 9B and 9C). It should be highlighted that the content of acetyl-CoA was increased by 40.4% and 69.0% compared to those of control at 12 h and 16 h, respectively (Fig 9B). In addition, ATP content was also enhanced significantly and the ATP/ADP ratio was increased by 2.28-fold compared to the control after 2 h addition of acetate, suggesting that acetate supplemented can increase energy metabolism in cells and produce more ATP for carotenoid production (Fig 9D). These results strongly suggested that a part of exogenous acetate can be converted to acetyl-CoA, then enter TCA cycle, ergosterol and fatty acids pathway, and fill up the pool of amino acids, TCA cycle and fatty acid and improve energy production in cells, which facilitate β-carotene formation. It also confirmed that acetate is an optimal carbon source for yeast to synthesize isoprenoid compounds [29].

## Discussion

To identify the potential factors limiting the formation of carotenoids at metabolic level, comparative metabolic analysis was conducted on β-carotene-producing recombinant yeast T73-63 and the parent strain T73-4 in this study. The obtained results showed that heterologous biosynthesis of carotenoids in *S*. *cerevisiae* significantly reduce intracellular metabolites pool (including amino acid, glycolysis and TCA cycle intermediates, fatty acid and energy metabolites) compared to the parent strain, especially in the rapid synthesis phase of carotenoids (Figs 3 and 4).

In line with our results, Lee et al. [30] also observed that reduced amino acid levels in the high carotenoid-producing *R*. *toruloides* as compared to the low-yield strain. Accordingly, Nam et al. [31] found that added specific amino acids such as glutamine, isoleucine, leucine, lysine, phenylalanine and threonine could effectively increase β-carotene production in recombinant *E*. *coli*. These results collectively suggested that amino acid is essential for carotenoids biosynthesis and enrichment of amino acids in cells is a potential way to improve the production of carotenoids. The intermediates in glycolysis and TCA cycle and fatty acids were also down-regulated in recombinant strain relative to the parent strain (Fig 4B and 4C). There is a strong correlation between the carbon source used and the level of carotenoid synthesis [26]. However, high glucose concentrations as carbon source could only improve cell growth while exhibited minimal pigment synthesis (Figs 5 and 6) due to the repression of glucose on genes expressions in mevalonate pathway [28]. In comparison, non-fermentable carbon sources are beneficial for carotenoids biosynthesis, including ethanol [32], glycerol [33,34], citrate [35] and succinate [10]. This is because these substances can be directly or indirectly converted to acetyl-CoA and flux into the carotenoid synthesis pathway [36]. Similarly, exogenous addition of fatty acid can promote carotenoid formation due to driving more acetyl-CoA entering carotenoid synthesis pathway [23]. We also found that the cofactors (NAD^+^, NADH, NADP^+^ and NADPH) and ATP level were reduced in T73-63 strain compared to T73-4 strain (Fig 4D), suggesting that heterologous carotenoid synthesis can consume more energy in yeast, and energy insufficiency might be the limited factor restricting further enhancement of carotenoid in the recombinant strain [5].

It is interesting to find that higher amount of trehalose was accumulated in recombinant strain T73-63. This is similar to the results of Martinez-Moya et al. [10]. The synthesis of trehalose is considered as a defense response of cells to a variety of stress conditions [10,37]. The up-regulation of trehalose in this case implied that heterologous biosynthesis of β-carotene in yeast can generate specific stress conditions to cells. This is correspondence to the conclusion of Verwaal et al. [16] that accumulation of carotenoids in *S*. *cerevisiae* could cause membrane stress and induce the toxicity within the cells. Based on the results, we proposed that accumulating trehalose might be a strategy used by cells to counteract the toxic effects of heterologous synthetic carotenoids. Thus, the amount of trehalose in cells might be positive with carotenoids concentration produced in yeast. This assumption could be partially verified by the observation of trehalose and β-carotene concentrations were simultaneously increased in the acetate addition experiment (Fig 9B). The detailed mechanism required to be further explored.

**Fig 10:**
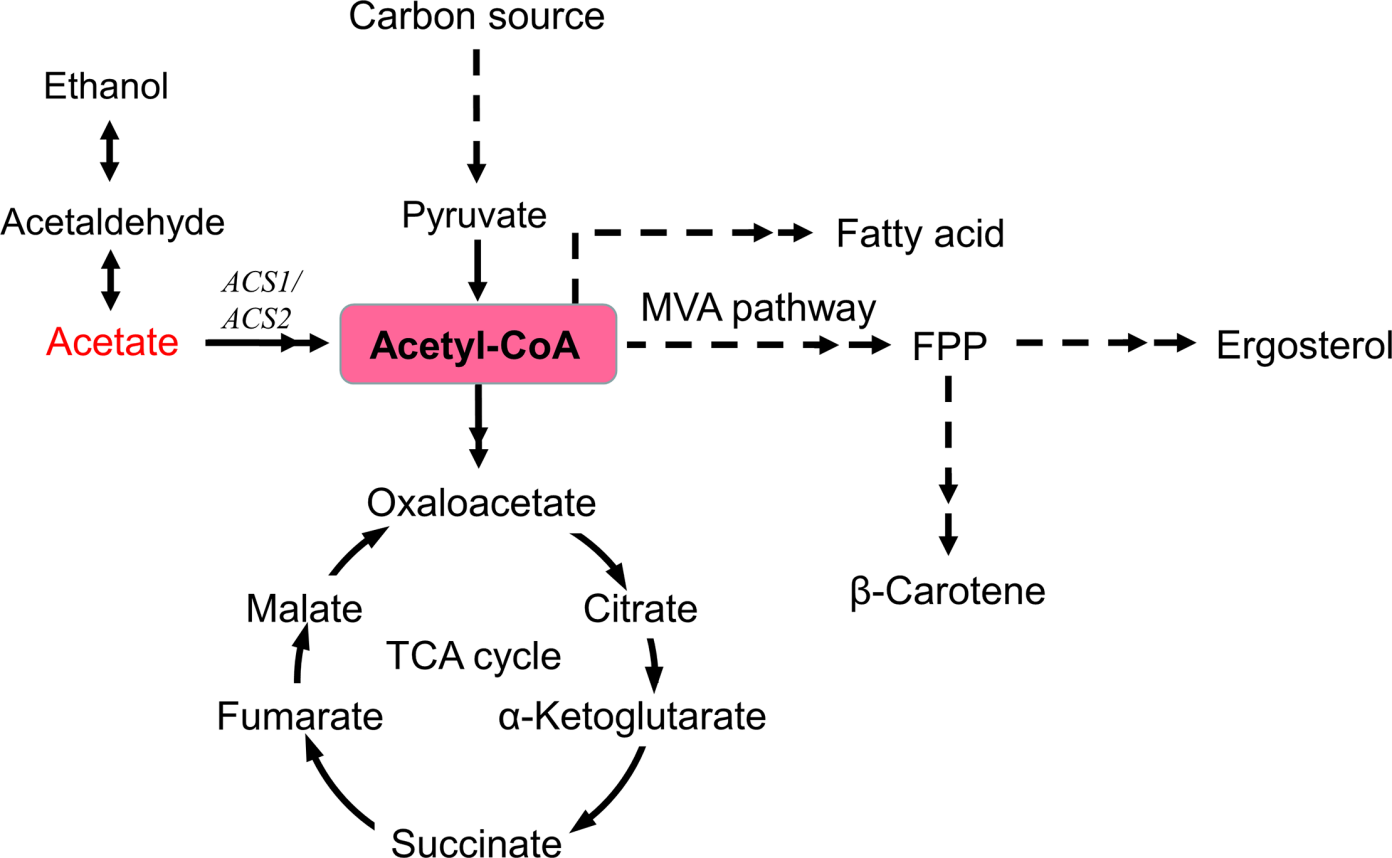
The conversion pathway of acetate into β-carotene in recombinant *S*. *cerevisiae*. Acetate addition are shown in red, central metabolite (acetyl-CoA) are shown in rose. The dashed lines represent multiple step reactions, double arrows indicate the enhancement of metabolic flow. *ACS1/ACS2*: Acetyl-CoA synthetase, FPP: farnesyl pyrophosphate, MVA pathway: mevalonate pathway.

Acetyl-CoA is a key molecule in central carbon metabolism in yeast, which is not only used for basic cellular functions such as energy metabolism, lipid metabolism and amino acid metabolism, but also serves as a precursor for forming isoprenoid compounds [38]. In *S*. *cerevisiae*, acetyl-CoA metabolism is highly compartmentalized and occurs in the cytosol, mitochondria, peroxisomes, and nucleus. Most acetyl-CoA is generated in mitochondria, however, isoprenoids compounds are produced by the mevalonate pathways that use cytosol acetyl-CoA, but *S*. *cerevisiae* lacks the machinery to export the mitochondrial acetyl-CoA to the cytosol [39]. Therefore, the supply of sufficient amounts of the precursor acetyl-CoA in the cytoplasm is crucial for isoprenoid compounds formation. To achieve this purpose, several metabolic engineering strategies have been carried out to boost the availability of cytosol acetyl-CoA in yeast, such as deletion of *Δypl062w *to increase lycopene [40], over-expression of *ALD6*, *acsSE L641P*, and *ADH2* to increase sesquiterpenes [41] and over-expression of *PDH* or *PDHm* to increase polyketide [42]. In the cytosol, acetyl-CoA is generated via direct activation of acetate in an ATP-dependent reaction by an acetyl-CoA synthetase (*ACS1* and *ACS2*). Besides over-expression of *ACS1* and (or) *ACS2* [43], increasing acetate concentration in medium can also raise intracellular acetyl-CoA pool (Fig 8B), although the conversion efficiency is limited due to the low activity and high energy input requirement of *ACS*. In this work, we found that supplying acetate can directly increase the content of acetyl-CoA and promote carotenoid biosynthesis, which was ascribed to more acetyl-CoA formation in cells (Fig 9B). Although the variation of *ACS* activity was not determined, the line of evidence has proved that the expression of *ACS1* can be derepression by the presence of acetate [44]. Previous study confirmed that the increment of carotenoid is usually accompanied by the high accumulation of fatty acid and ergosterol in cells due to both sharing the common precursor acetyl-CoA [29]. The analysis of metabolomics profiles confirmed this result because it was found that fatty acids and ergosterol contents were simultaneously increased by acetate addition, which can well prove the conclusion that more acetyl-CoA are produced and simultaneously fluxed into the fatty acids and mevalonate pathway (Fig 10). In addition, the increased contents of cofactors (NAD^+^, NADH, NADP^+^ and NADPH) and ATP level can also partially contribute to the improvement of β-carotene in acetate addition.

Acetic acid is a by-product produced by *S*. *cerevisiae* fermentation, which is also an inhibitor of yeast growth in lignocellulosic hydrolysates [45]. To increase the tolerance of yeast to acetic acid, engineered acetate metabolism to transform to acetyl-CoA by overexpression of *ACS2* is proved to be a feasible way [46]. Therefore, overexpression of *ACS* combined with rational exogenous addition of acetate might be a feasible way to increase acetyl-CoA derived products. It should be mentioned that in this study, the resistant ability of the recombinant strain T73-63 to acetate is relatively high (reaching 10 g/l) compared to other registered strains [40]. This could be due to the fact that the parent strain T73-4 is industrial wine yeast, which can well adapt to harsh condition of grape juice contains high sugar concentrations (150–260 g/l) and low pH (2.9–3.6), and has a high ability of assimilating acetate [47]. Hence, using the yeast strain with high resistance to environmental stress, especially high tolerance to acetate as candidate strain for synthesizing acetyl-CoA derived products is a better choice.

In summary, with the help of metabolomics data analysis, we found that heterogeneous biosynthesis of carotenoid in *S*. *cerevisiae* can redirect most of intermediate metabolites from glycolysis, TCA cycle and fatty acid pathway into carotenoid branch, which result in insufficiency of intracellular metabolites, cofactors and ATP level. Exogenous addition of acetate in exponential phase could partially restore most intracellular metabolites, and result in marked increment of acetyl-CoA, cofactor content and ATP level, which provides sufficient precursor and energy for carotenoid biosynthesis. Therefore, it can be concluded that maintaining the normal level of intracellular metabolites is essential for carotenoid biosynthesis in microorganisms. To relieve this limitation, increasing the transformation of acetate to acetyl-CoA by rational supplementation of acetate or (and) modification of *ACS* activity with genetic engineering might a potential strategy to increase the production of isoprenoid compounds when using *S*. *cerevisiae* as a cell factory.

## Supporting information

S1 Fig **Changes of cell growth (A), specific growth rates (B), sugar consumption (C), specific sugar consumption rates (D), ethanol concentration (E) and acetic acid concentration (F) of recombinant *S*. *cerevisiae* T73-63 (squares; curve 1) and T73-63 supplemented with 10 g/l acetate at 0 h (circles; curve 2) and 10 h (triangles; curve 3).** Values represent the average of three independent cultures, and error bars correspond to the standard deviation (*P*< 0.05, Student’s *t* test).
(TIF)

S1 Table List of all differential metabolites in parent strain T73-4 and recombinant *S*. *cerevisiae* T73-63 at 8, 12 and 16 h. (DOC)

S2 Table List of all differential metabolites in strain T73-63 and strain T73-63 with acetate supplementation at 10 h. (DOC)
