# Supplementary figures and images for: Comparative metabolomics profiling of engineered Saccharomyces cerevisiae lead to a strategy that improving β-carotene production by acetate supplementation
Source: PLoS One. 2017 Nov 21;12(11):e0188385. doi: 10.1371/journal.pone.0188385 (PMC5697841; doi:10.1371/journal.pone.0188385)

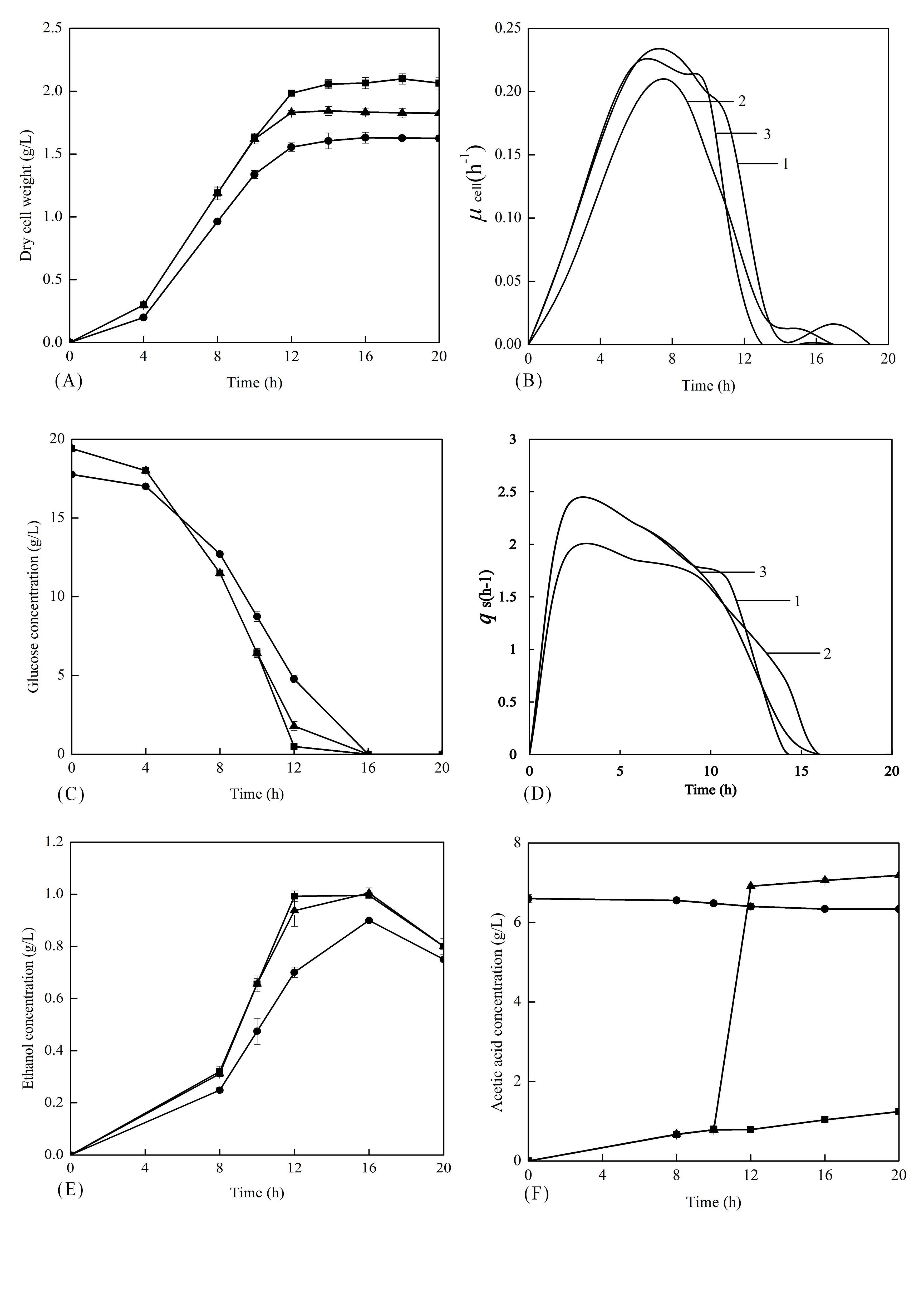

Supplement: S1 Fig — Changes of cell growth (A), specific growth rates (B), sugar consumption (C), specific sugar consumption rates (D), ethanol concentration (E) and acetic acid concentration (F) of recombinant S. cerevisiae T73-63 (squares; curve 1) and T73-63 supplemented with 10 g/l acetate at 0 h (circles; curve 2) and 10 h (triangles; curve 3). Values represent the average of three independent cultures, and error bars correspond to the standard deviation (P< 0.05, Student’s t test). (TIF) [file pone.0188385.s001.tif]
