# Supplementary material for: Comparative metabolomics profiling of engineered Saccharomyces cerevisiae lead to a strategy that improving β-carotene production by acetate supplementation
Source: PLoS One. 2017 Nov 21;12(11):e0188385. doi: 10.1371/journal.pone.0188385 (PMC5697841; doi:10.1371/journal.pone.0188385)
Supplement: S1 Table — (DOC) [file pone.0188385.s002.doc]

**S1 Table. List of all differential metabolites in parent strain T73-4 and recombinant *S. cerevisiae* T73-63 at 8, 12 and 16 h.**

| Sample  Metabolites | T73-4 (8h) | T73-63 (8h) | T73-4 (12h) | T73-63 (12h) | T73-4 (16h) | T73-63 (16h) |
| --- | --- | --- | --- | --- | --- | --- |
| Ala | 25.048±2.449 | 5.013±0.333 | 13.593±0.302 | 13.134±1.215 | 13.682±0.440 | 15.154±1.341 |
| Arg | 6.693±1.326 | 3.622±0.101 | 3.381±0.233 | 3.666±0.759 | 4.257±0.901 | 3.411±0.510 |
| Asn | 0.040±0.004 | 0.018±0.000 | 0.031±0.001 | 0.040±0.001 | 0.035±0.002 | 0.087±0.029 |
| Asp | 13.980±1.754 | 3.365±0.305 | 6.332±0.333 | 7.009±0.865 | 7.193±0.712 | 7.446±0.222 |
| Cys | 0.080±0.007 | 0.031±0.003 | 0.122±0.009 | 0.522±0.143 | 1.577±0.152 | 2.936±0.481 |
| Gln | 1.547±0.245 | 0.796±0.082 | 0.562±0.016 | 1.561±0.076 | 0.997±0.378 | 2.828±0.386 |
| Glu | 23.785±3.161 | 6.785±0.634 | 19.958±0.971 | 14.816±1.743 | 29.865±4.180 | 13.326±1.097 |
| Gly | 16.632±1.543 | 5.055±0.717 | 4.987±0.143 | 8.918±0.568 | 4.631±0.143 | 8.629±0.747 |
| His | 1.978±0.392 | 1.016±0.058 | 1.172±0.040 | 1.603±0.282 | 1.562±0.075 | 1.690±0.185 |
| Ile | 9.517±1.192 | 2.171±0.078 | 4.078±0.142 | 3.871±0.108 | 5.548±0.758 | 5.713±0.251 |
| Leu | 12.938±1.696 | 3.415±0.082 | 5.216±0.262 | 5.490±0.132 | 6.823±1.087 | 8.097±0.228 |
| Lys | 12.524±2.135 | 6.095±0.233 | 7.081±0.304 | 8.225±1.435 | 8.272±0.533 | 10.745±1.231 |
| Met | 2.847±0.360 | 0.764±0.027 | 1.172±0.057 | 0.996±0.013 | 1.348±0.266 | 1.418±0.045 |
| Phe | 6.066±0.729 | 1.684±0.044 | 2.532±0.166 | 2.927±0.104 | 3.146±0.524 | 4.241±0.176 |
| Pro | 6.416±0.791 | 1.951±0.123 | 3.170±0.044 | 4.065±0.341 | 4.590±0.244 | 5.444±0.404 |
| Ser | 17.157±1.668 | 0.474±0.277 | 10.314±0.389 | 7.205±0.858 | 8.079±1.164 | 8.108±2.863 |
| Thr | 9.399±1.152 | 2.771±0.058 | 3.846±0.221 | 4.843±0.136 | 3.319±2.864 | 5.539±0.415 |
| Trp | 0.670±0.087 | 0.254±0.011 | 0.264±0.010 | 0.409±0.011 | 0.317±0.055 | 0.553±0.020 |
| Tyr | 3.852±0.493 | 1.186±0.036 | 1.655±0.115 | 1.946±0.017 | 1.853±0.68 | 2.860±0.093 |
| Val | 12.305±1.556 | 2.667±0.183 | 5.119±0.246 | 4.584±0.204 | 5.915±0.798 | 6.658±0.387 |
| L-lactic acid | 4.928±0.065 | 4.614±0.195 | 5.750±0.082 | 4.850±0.079 | 5.699±0.172 | 5.066±0.249 |
| glycolic acid | 3.470±0.067 | 3.659±0.123 | 3.550±0.095 | 4.278±0.069 | 3.969±0.111 | 4.162±0.072 |
| oxalic acid | 4.819±0.160 | 4.636±0.146 | 5.006±0.103 | 5.184±0.073 | 4.986±0.078 | 5.039±0.085 |
| acetohydroxamic acid | 4.344±0.051 | 4.605±0.044 | 4.594±0.144 | 4.820±0.034 | 4.581±0.095 | 4.807±0.037 |
| malonic acid | 4.255±0.201 | 4.425±0.079 | 4.445±0.172 | 4.805±0.120 | 4.503±0.122 | 4.521±0.217 |
| phosphoric acid | 6.506±0.181 | 5.819±0.079 | 6.870±0.104 | 5.977±0.132 | 6.890±0.025 | 5.866±0.060 |
| acetic acid | 5.461±0.152 | 2.526±0.163 | 5.754±0.106 | 2.964±0.106 | 6.187±0.030 | 3.160±0.137 |
| citric acid | 4.376±0.195 | 2.925±0.089 | 4.685±0.147 | 3.462±0.263 | 4.842±0.020 | 3.525±0.149 |
| hydrocinnamic acid | 3.921±0.079 | 4.180±0.137 | 4.189±0.148 | 4.327±0.082 | 4.284±0.044 | 4.396±0.061 |
| capric acid | 2.576±0.223 | 2.661±0.059 | 1.746±1.514 | 3.009±0.259 | 2.781±0.158 | 3.059±0.435 |
| iminodiacetic acid | 3.874±0.162 | 3.408±0.177 | 4.091±0.131 | 4.305±0.119 | 4.224±0.074 | 4.317±0.177 |
| gamma-aminobutyric acid | 4.204±0.406 | 3.502±0.145 | 5.056±0.258 | 5.182±0.065 | 5.537±0.133 | 5.664±0.087 |
| 4-guanidinobutyric acid | 4.173±0.095 | 4.315±0.159 | 4.343±0.095 | 4.461±0.079 | 4.373±0.036 | 4.341±0.146 |
| 5-aminovaleric acid | 4.583±0.199 | 4.805±0.104 | 4.970±0.177 | 5.147±0.057 | 5.042±0.024 | 5.223±0.163 |
| lauric acid | 4.012±0.074 | 3.908±0.168 | 4.068±0.068 | 4.104±0.041 | 4.072±0.080 | 3.969±0.176 |
| myristic acid | 4.203±0.060 | 4.335±0.114 | 4.255±0.105 | 4.390±0.015 | 4.155±0.164 | 4.250±0.169 |
| eicosapentaenoic acid | 1.685±1.461 | 0.000±0.000 | 2.831±0.139 | 4.184±0.042 | 3.136±0.094 | 3.878±0.018 |
| 2,3-dihydroxybenzoic acid | 3.445±0.025 | 3.709±0.189 | 3.492±0.181 | 3.822±0.098 | 3.661±0.036 | 3.728±0.118 |
| nicotinic acid | 4.511±0.135 | 4.105±0.175 | 4.736±0.152 | 4.844±0.052 | 4.917±0.041 | 4.706±0.062 |
| palmitoleic acid | 5.032±0.105 | 2.282±0.128 | 5.300±0.122 | 3.499±0.033 | 5.311±0.074 | 4.383±0.101 |
| palmitic acid | 5.442±0.034 | 4.718±0.080 | 5.607±0.132 | 5.150±0.076 | 5.901±0.100 | 5.508±0.129 |
| oleic acid | 3.430±0.134 | 1.623±0.086 | 4.760±0.133 | 2.872±0.058 | 5.811±0.137 | 3.767±0.088 |
| 1-stearoyl-rac-glycerol | 3.193±0.281 | 2.383±0.112 | 3.347±0.152 | 2.704±0.026 | 3.265±0.072 | 3.078±0.045 |
| pyruvic acid | 4.254±0.067 | 3.644±0.093 | 4.556±0.077 | 4.358±0.061 | 5.734±0.067 | 4.415±0.141 |
| succinic acid | 5.219±0.170 | 4.452±0.310 | 5.935±0.011 | 5.035±0.009 | 5.158±0.035 | 4.406±0.134 |
| glycerol 1-phosphate | 4.287±0.263 | 4.155±0.104 | 4.919±0.066 | 4.516±0.141 | 5.755±0.084 | 4.664±0.078 |
| pelargonic acid | 3.439±0.074 | 3.576±0.144 | 3.572±0.135 | 3.545±0.117 | 3.649±0.115 | 3.667±0.041 |
| beta-sitosterol | 1.049±0.817 | 3.393±0.090 | 3.535±0.289 | 3.593±0.051 | 3.498±0.128 | 5.716±0.070 |
| myo-inositol | 5.230±0.072 | 5.413±0.166 | 5.395±0.111 | 5.680±0.034 | 5.680±0.105 | 5.716±0.070 |
| galactinol | 5.093±0.196 | 4.774±0.090 | 5.466±0.147 | 5.342±0.078 | 5.653±0.024 | 5.298±0.101 |
| ethanolamine | 4.781±0.057 | 5.029±0.105 | 4.921±0.216 | 5.153±0.021 | 4.972±0.080 | 5.100±0.049 |
| D-threitol | 2.793±0.134 | 2.967±0.244 | 3.031±0.125 | 3.924±0.052 | 3.157±0.025 | 3.856±0.075 |
| xylitol | 5.502±0.055 | 5.636±0.065 | 5.668±0.107 | 5.956±0.020 | 5.776±0.072 | 5.726±0.101 |
| glycerol | 5.128±0.053 | 2.866±0.151 | 5.206±0.188 | 4.220±0.038 | 5.417±0.271 | 3.833±0.099 |
| D-lyxose | 2.984±0.093 | 2.552±0.190 | 2.806±0.257 | 3.913±0.011 | 3.558±0.155 | 3.673±0.223 |
| ribose | 4.869±0.250 | 4.457±0.141 | 5.217±0.153 | 5.352±0.035 | 5.236±0.143 | 4.986±0.104 |
| fructose | 5.520±0.020 | 2.447±0.209 | 5.763±0.089 | 4.464±0.204 | 6.235±0.099 | 4.461±0.099 |
| D-glucose | 4.935±0.015 | 3.093±0.222 | 5.207±0.061 | 3.847±0.201 | 6.701±0.120 | 4.662±0.223 |
| D-allose | 4.507±0.054 | 3.296±0.290 | 4.767±0.071 | 5.548±0.103 | 5.357±0.080 | 5.260±0.089 |
| galactose | 4.019±0.163 | 3.155±0.095 | 4.896±0.045 | 3.764±0.099 | 5.426±0.098 | 3.980±0.079 |
| beta-gentiobiose | 4.772±0.516 | 4.604±0.174 | 5.252±0.123 | 5.193±0.183 | 5.523±0.023 | 5.429±0.077 |
| D-trehalose | 3.512±0.257 | 3.480±0.084 | 4.634±0.257 | 6.201±0.239 | 5.002±0.175 | 6.223±0.110 |
| isomaltose | 4.821±0.608 | 3.451±0.174 | 6.028±0.613 | 4.567±0.161 | 6.294±1.077 | 4.653±0.050 |
| Hexadecene | 3.491±0.078 | 3.794±0.240 | 3.679±0.154 | 4.004±0.186 | 3.780±0.055 | 3.792±0.217 |
| 2-piperidone | 5.041±0.019 | 5.244±0.047 | 5.257±0.121 | 5.273±0.149 | 5.300±0.030 | 5.396±0.015 |
| phenethylamine | 4.498±0.197 | 4.742±0.083 | 4.868±0.133 | 5.088±0.062 | 4.906±0.086 | 5.097±0.200 |
| galactosamine | 3.476±0.037 | 1.934±0.708 | 3.279±0.015 | 3.647±0.058 | 3.547±0.256 | 2.201±0.941 |
| benzoin | 3.048±1.091 | 1.711±0.484 | 4.471±0.364 | 4.334±0.108 | 4.661±0.193 | 5.256±0.110 |
| xanthotoxin | 4.999±0.074 | 5.331±0.046 | 5.233±0.136 | 5.417±0.028 | 5.184±0.175 | 5.388±0.023 |
| uracil | 4.376±0.195 | 3.925±0.089 | 4.685±0.147 | 4.462±0.263 | 4.842±0.020 | 4.525±0.149 |
| 2-hydroxypyridine | 4.710±0.052 | 4.919±0.064 | 4.975±0.146 | 5.210±0.054 | 5.171±0.026 | 5.183±0.163 |
| 3-hydroxypyridine | 4.077±0.067 | 4.253±0.086 | 4.295±0.167 | 4.583±0.022 | 4.380±0.042 | 4.525±0.150 |
| 2,3-dihydroxypyridine | 3.676±0.072 | 3.873±0.070 | 3.858±0.088 | 3.989±0.126 | 4.004±0.027 | 4.057±0.127 |
| ADP | 5.179±0.055 | 1.828±0.017 | 5.374±0.014 | 5.114±0.083 | 5.244±0.39 | 2.929±0.117 |
| ATP | 3.115±0.061 | 0.793±0.019 | 4.192±0.131 | 3.8±0.013 | 4.482±0.178 | 1.735±0.035 |
| NADP^+^ | 3.982±0.11 | 2.794±0 | 4.111±0.182 | 2.87±0.015 | 4.152±0.259 | 2.3±0.181 |
| NAD^+^ | 4.161±0.091 | 2.574±0.039 | 4.062±0.201 | 3.497±0.607 | 3.768±0.062 | 3.582±0.213 |
| NADPH | 4.463±0.617 | 0.631±0.319 | 1.356±0.633 | 0±0 | 0.082±0.016 | 0.592±0.434 |
| NADH | 2.046±0.212 | 1.39±0.137 | 2.748±0.642 | 0.125±0.023 | 0.663±0.063 | 0.222±0.035 |
| CoA | 3.217±0.098 | 2.915±0.037 | 5.06±0.1 | 2.688±0.242 | 3.266±1.148 | 3.224±0.48 |
| Acetyl-CoA | 4.629±0.11 | 3.364±0.163 | 2.655±0.162 | 2.75±0.096 | 1.919±0.04 | 2.137±0.095 |
| Ergosterol | 4.3±0.11 | 2.9±0.263 | 4.2±0.006 | 3.3±0.196 | 3.9±0.004 | 3.1±0.095 |
