# Supplementary material for: Comparative metabolomics profiling of engineered Saccharomyces cerevisiae lead to a strategy that improving β-carotene production by acetate supplementation
Source: PLoS One. 2017 Nov 21;12(11):e0188385. doi: 10.1371/journal.pone.0188385 (PMC5697841; doi:10.1371/journal.pone.0188385)
Supplement: S2 Table — (DOC) [file pone.0188385.s003.doc]

**S2 Table. List of all differential metabolites in strain T73-63 and strain T73-63 with acetate supplementation at 10 h.**

| Sample    Metabolites | Glu (12 h) | Glu+AceNa (12 h) | Glu (16 h) | Glu+AceNa (16 h) |
| --- | --- | --- | --- | --- |
| Ala | 3.247±0.110 | 4.757±0.309 | 4.698±0.080 | 5.107±0.056 |
| Arg | 3.582±0.007 | 3.203±0.088 | 4.898±0.059 | 5.042±0.324 |
| Asn | 0.585±0.003 | 4.683±0.008 | 4.260±0.005 | 5.698±0.043 |
| Asp | 4.673±0.044 | 5.227±0.318 | 4.967±0.148 | 5.513±0.154 |
| Cys | 3.800±0.004 | 3.800±0.087 | 3.800±0.129 | 3.800±0.026 |
| Gln | 2.079±0.096 | 3.816±0.020 | 3.781±0.100 | 4.513±0.363 |
| Glu | 3.941±0.003 | 5.179±0.313 | 4.449±0.005 | 5.300±0.541 |
| Gly | 3.773±0.101 | 4.970±0.252 | 5.383±0.447 | 5.699±0.234 |
| His | 4.027±0.027 | 4.522±0.022 | 5.205±0.037 | 5.913±0.083 |
| Ile | 2.825±0.058 | 4.393±0.032 | 4.293±0.094 | 4.741±0.013 |
| Leu | 3.050±0.007 | 4.540±0.191 | 4.500±0.003 | 5.000±0.171 |
| Lys | 2.808±0.203 | 4.320±0.172 | 4.429±0.068 | 4.976±0.171 |
| Met | 3.432±0.028 | 4.884±0.050 | 4.752±0.037 | 5.200±0.025 |
| Phe | 1.947±0.048 | 2.923±0.060 | 2.895±0.041 | 3.194±0.053 |
| Pro | 2.824±0.005 | 4.205±0.349 | 4.135±0.107 | 4.403±0.294 |
| Ser | 3.450±0.075 | 4.230±0.370 | 4.360±0.250 | 4.840±0.096 |
| Thr | 3.412±0.012 | 4.922±0.166 | 4.833±0.106 | 5.282±0.165 |
| Trp | 3.700±0.013 | 5.747±0.008 | 5.487±0.013 | 6.316±0.042 |
| Tyr | 2.974±0.067 | 4.550±0.043 | 4.450±0.037 | 4.894±0.077 |
| Val | 2.540±0.019 | 3.848±0.155 | 3.694±0.055 | 4.071±0.068 |
| 2-piperidone | 3.437±0.004 | 3.527±0.016 | 3.580±0.116 | 3.769±0.001 |
| 2-hydroxypyridine | 4.623±0.036 | 4.652±0.013 | 4.646±0.034 | 4.688±0.022 |
| pyruvic acid | 4.524±0.005 | 4.631±0.016 | 4.608±0.003 | 4.677±0.015 |
| L-(+) lactic acid | 3.695±0.062 | 3.523±0.035 | 3.373±0.076 | 3.419±0.028 |
| glycolic acid | 4.902±0.023 | 5.105±0.009 | 5.072±0.024 | 5.118±0.004 |
| oxalic acid | 4.912±0.004 | 5.265±0.028 | 5.316±0.057 | 5.352±0.005 |
| acetohydroxamic acid | 4.140±0.016 | 4.241±0.017 | 4.210±0.028 | 4.263±0.020 |
| 3-hydroxypyridine | 3.694±0.159 | 3.577±0.474 | 3.930±0.003 | 4.055±0.093 |
| malonic acid | 4.470±0.028 | 4.483±0.011 | 4.433±0.015 | 4.588±0.155 |
| ethanolamine | 4.341±0.003 | 4.410±0.060 | 4.379±0.100 | 4.537±0.005 |
| phosphoric acid | 5.510±0.047 | 5.670±0.061 | 5.602±0.012 | 5.595±0.007 |
| nicotinic acid | 3.810±0.025 | 3.811±0.190 | 3.776±0.265 | 3.919±0.214 |
| succinic acid | 4.665±0.818 | 4.396±0.218 | 4.422±0.248 | 4.446±0.178 |
| 2,3-dihydroxypyridine | 3.398±0.012 | 3.465±0.037 | 3.427±0.071 | 3.484±0.062 |
| glyceric acid | 4.790±0.027 | 4.994±0.013 | 4.999±0.060 | 4.973±0.068 |
| uracil | 5.004±0.020 | 5.197±0.005 | 5.218±0.060 | 5.167±0.050 |
| citraconic acid | 5.004±0.020 | 5.197±0.005 | 5.218±0.060 | 5.167±0.050 |
| pelargonic acid | 3.047±0.078 | 3.158±0.023 | 3.147±0.025 | 3.297±0.070 |
| hydrocinnamic acid | 4.214±0.141 | 4.564±0.217 | 4.742±0.085 | 4.588±0.238 |
| capric acid | 3.019±0.005 | 3.032±0.010 | 3.174±0.221 | 3.068±0.060 |
| iminodiacetic acid | 2.956±0.037 | 3.318±0.001 | 3.458±0.081 | 3.536±0.066 |
| phenethylamine | 4.883±0.117 | 5.361±0.007 | 5.473±0.075 | 5.430±0.020 |
| D-threitol | 2.798±0.116 | 2.720±0.064 | 3.001±0.031 | 2.987±0.164 |
| gamma-aminobutyric acid | 4.885±0.116 | 5.363±0.008 | 5.473±0.076 | 5.431±0.019 |
| 4-guanidinobutyric acid | 2.031±0.422 | 2.506±0.033 | 2.615±0.042 | 2.726±0.038 |
| hexadecene | 3.986±0.086 | 3.988±0.000 | 3.880±0.006 | 3.999±0.017 |
| 5-aminovaleric acid | 3.112±0.080 | 3.277±0.057 | 3.371±0.024 | 3.420±0.004 |
| lauric acid | 3.582±0.010 | 3.479±0.074 | 3.736±0.034 | 3.599±0.014 |
| 2,3-dihydroxybenzoic acid | 2.978±0.044 | 3.094±0.061 | 2.996±0.180 | 3.214±0.054 |
| D-lyxose | 5.052±0.019 | 5.172±0.022 | 5.126±0.025 | 5.206±0.124 |
| ribose | 5.062±0.017 | 5.162±0.012 | 5.150±0.007 | 5.191±0.119 |
| xylitol | 5.846±0.010 | 5.880±0.007 | 5.876±0.024 | 5.886±0.025 |
| glycerol 1-phosphate | 4.561±0.009 | 4.906±0.034 | 5.033±0.138 | 4.999±0.038 |
| myristic acid | 3.612±0.061 | 3.774±0.022 | 3.865±0.046 | 3.929±0.061 |
| galactosamine | 6.245±0.002 | 6.096±0.049 | 6.201±0.005 | 5.995±0.029 |
| benzoin | 3.422±0.914 | 5.142±0.042 | 5.225±0.144 | 5.242±0.121 |
| fructose | 4.525±0.022 | 4.338±0.065 | 4.564±0.044 | 4.218±0.011 |
| D-glucose | 6.281±0.000 | 6.105±0.065 | 6.226±0.004 | 6.008±0.033 |
| D-mannitol | 5.398±0.002 | 5.253±0.058 | 5.349±0.006 | 5.153±0.037 |
| palmitoleic acid | 5.141±0.026 | 5.360±0.058 | 5.301±0.006 | 5.312±0.050 |
| palmitic acid | 5.065±0.004 | 5.161±0.003 | 5.170±0.083 | 5.163±0.085 |
| myo-inositol | 5.425±0.017 | 5.685±0.019 | 5.742±0.048 | 5.650±0.079 |
| D-allose | 4.566±0.038 | 4.397±0.018 | 4.752±0.091 | 4.288±0.113 |
| oleic acid | 4.268±0.045 | 4.560±0.025 | 4.488±0.077 | 4.142±0.441 |
| xanthotoxin | 4.499±0.009 | 4.574±0.005 | 4.592±0.075 | 4.590±0.083 |
| eicosapentaenoic acid | 3.358±0.012 | 4.894±0.049 | 5.027±0.076 | 4.851±0.063 |
| lactulose | 4.530±0.186 | 5.035±0.039 | 5.162±0.066 | 4.988±0.073 |
| 1-stearoyl-rac-glycerol | 2.723±0.293 | 3.348±0.011 | 3.397±0.077 | 3.362±0.021 |
| beta-gentiobiose | 4.547±0.048 | 4.841±0.152 | 4.968±0.125 | 4.919±0.037 |
| D-(+) trehalose | 4.548±0.058 | 4.844±0.167 | 4.970±0.138 | 4.915±0.039 |
| isomaltose | 3.748±0.051 | 3.923±0.130 | 3.909±0.108 | 3.931±0.072 |
| galactinol | 5.023±0.037 | 5.097±0.036 | 5.371±0.017 | 5.016±0.045 |
| ADP | 5.114±0.083 | 6.093±0.326 | 2.929±0.117 | 3.411±0.11 |
| ATP | 2.223±0.013 | 6.015±0.322 | 0.867±0.035 | 1.291±0.036 |
| NADP^+^ | 2.87±0.015 | 3.143±0.138 | 2.3±0.181 | 2.109±0.153 |
| NAD^+^ | 3.497±0.607 | 3.352±0.172 | 3.582±0.213 | 3.289±0.401 |
| NADPH | 0±0 | 5.408±0.92 | 0.592±0.434 | 0.436±0.267 |
| NADH | 0.125±0.023 | 5.886±0.769 | 0.222±0.035 | 1.312±0.328 |
| CoA | 2.688±0.242 | 1.438±0.011 | 3.224±0.48 | 1.135±0.11 |
| Acetyl-CoA | 3.613±0.196 | 5.07±0.061 | 2.137±0.095 | 3.612±0.135 |
| Ergosterol | 3.3±0.196 | 4.95±0.213 | 3.1±0.095 | 4.6±0.172 |

Abbreviations: Glu: glucose, AceNa: sodium acetate
